# The Transformative Nature of Transparency in Research Funding

**DOI:** 10.1371/journal.pbio.1002027

**Published:** 2014-12-30

**Authors:** Daniel Mietchen

**Affiliations:** Museum für Naturkunde Berlin, Leibniz-Institut für Evolutions- und Biodiversitätsforschung, Berlin, Germany

## Abstract

Central to research funding are grant proposals that researchers send in to potential funders for review, in the hope of approval. A survey of policies at major research funders found that there is room for more transparency in the process of grant review, which would strengthen the case for the efficiency of public spending on research. On that basis, debate was invited on which transparency measures should be implemented and how, with some concrete suggestions at hand. The present article adds to this discussion by providing further context from the literature, along with considerations on the effect size of the proposed measures. The article then explores the option of opening to the public key components of the process, makes the case for pilot projects in this area, and sketches out the potential that such measures might have to transform the research landscape in those areas in which they are implemented.

## Abstract 2

This Perspective extends an ongoing debate on transparency in research funding, advocating the exploration of more radical approaches.

## Transparency in Research Funding?

In this issue of *PLOS Biology*, Gurwitz et al. [1] (subsequently referred to as GMK) report on a survey of transparency at major funders of biomedical research. They looked at what information funders make public about assessment procedures and funded proposals, as detailed in their Table 1.

On that basis, GMK discuss the merits of adding transparency to the grant proposal review process in one of two ways: First, in what they refer to as the “incremental” approach, individual components of the process would be made more open. Focusing on components that “should not do any harm on the evaluation procedure at all,” they suggest that funders publish

1. (once funding decisions have been announced for a call) a list of the members of that call's review panels, along with a cumulative list of external reviewers;
2. statements within the application about the expected impact of the proposed research;
3. the final reports of funded projects.

The second approach—termed the “radical” one—is about opening up the review process as a whole, rather than selected parts thereof. The authors provide some practical considerations as to why that might be useful:

- It addresses reviewer fatigue.
- Published peer reviews can be helpful to readers.
- It would promote rather than inhibit collaboration between researchers.
- It would allow more public participation in research.

They suggest the radical approach may be “quite transformative” (or “sweeping”) in terms of both scholarly communication and public participation in research. They caution that the current research system and associated evaluation procedures are not set up for such radical changes and then conclude by inviting debate on “which transparency measures to put in place, and how” [1].

In the following, I would like to follow this invitation by putting forth some thoughts on an open research funding system, its transformative nature, and how we might be able to pave the way to get there.

## Where Is the Evidence?

The most comprehensive meta-analysis of grant peer review to date is a 2007 paper by Demicheli and Di Pietrantonj (subsequently referred to as D&D) [2]. GMK quote one of its key statements: “There is little empirical evidence on the effects of grant giving peer review.” This is worth reading again, with an appreciation of how central the peer review of grant proposals has been—and continues to be—to our research funding system.

D&D titled their article “Peer review for improving the quality of grant applications,” which is interesting from a transparency perspective. Peer review can only improve the quality of grant applications if there is some form of feedback between the proposal and the review process, either within one round of submissions, across rounds, or across funders. Such feedback would certainly have a much wider reach (some would say “impact”) if it were public.

It is thus no surprise that one of the main conclusions by D&D is that “[a]ttempts to improve the efficiency and the transparency of the process and actions encouraging innovative ideas should be implemented and evaluated”—the other is that “[e]xperimental studies assessing the effects of grant-giving peer review on…funded research are urgently needed” [2].

Similar thoughts have been expressed in [3]: “[i]t is time to turn the scientific method on ourselves…by subjecting proposed reforms to a prospective, randomized, controlled experiment.” This idea makes intuitive sense, but it still does not tell us where to start with introducing transparency to this process.

## Effect Size

Let's assume that some funding agencies would like to open up their procedures and opt to implement the proposed incremental changes outlined above. How long would it take before any potential effects could be measured?

If, on the other hand, they were to implement some more profound (or “radical”) changes, shouldn't that result in larger effects that could be more readily observed and could inform relevant policy work earlier? Perhaps such an experiment works best under some controlled conditions—what might these conditions look like?

GMK identified “at least three” parameters suitable for incremental change [1]. In light of the complexity of the research funding system, it is not difficult to come up with further candidates by decomposing the funding process into its components. For instance, D&D have looked at different ways of processing submissions, of getting internal and external reviews, of making decisions based on those reviews, and of providing feedback to submitters. Other changes worth considering could include more transparency about the way funding calls or eligibility criteria are being designed, or to make public (and ideally machine readable) the data management plans now increasingly required for new proposals. Much of this could perhaps be engineered in a way that obeys the “no harm” condition put forth by GMK, but the magnitude of effects expected from any such incremental changes is not obvious.

What if final reports were public, as GMK suggest? Would they be useful without the context of the original proposals?

## Opening up Research Proposals

Now consider publishing the proposals themselves, assuming for the sake of argument that legal issues—e.g., as to who has the right to publish them—are no showstopper. GMK labeled this in their Box 2 as one of two “radical transparency measures” deemed “premature to recommend their open publication by default,” adding that they “would welcome small-scale experimentation in this area” [1]. The publication of the proposals could happen at various points in time, e.g.,

1. some years after a project has ended,
2. along with the final reports for a project,
3. at the beginning of a project,
4. at the point of announcing funding decisions,
5. upon submission to the funder,
6. during the drafting phase.

Knowing that many researchers have little attention to spare outside of their usual workflows, few of them would be expected to systematically go through past proposals on the basis of which funds have already been spent (scenarios 1 and 2 in the list above) or at least allocated (scenarios 3 and 4).

The situation is different if funds have not been allocated yet: scenario 5 may be useful for researchers to compare proposals and to gauge the likelihood of their own being funded. Furthermore, it might provide a basis for reaching out to potential collaborators for other proposals they are working on (or for the next stage, in case of multi-stage procedures). Going down the list, the potential for collaboration increases and is greatest in scenario 6 [4],[5]. It can be enhanced further by providing a public version history and allowing comments, edits, and forking or other kinds of reuse [6]. According to what GMK have framed as the “conventional wisdom” that sharing research before formal publication “would conflict with researchers' interests” [1], scenario 6 would seem to be the least compatible with the “no harm” approach. On the other hand, it is hard to imagine how scenarios 1 and 2 might cause any harm at all for successful proposals. Even for unsuccessful ones from the same call, the effects should be minimal after so much time has gone by. Either way, there is very little data on any of these scenarios, so “small-scale experimentation in this area” would indeed seem like a good option to find out more.

The concept of discussing proposals in public is not entirely new: participatory budgeting [7] and grantmaking [8] have been explored in a number of societal contexts, whereas large-scale research infrastructures like the Large Hadron Collider [9] have long been planned in public, and at the lower end of the budget scale, recent years have seen a first wave of crowdfunding initiatives for research [10], with proposals being public by default. At the typical scale of projects funded by the agencies surveyed by GMK, the default is certainly not to publish proposals, though a few individuals and groups do it nonetheless [11],[12]. While budgets are interesting, they depend on a number of non-scientific parameters, so making them public is less essential than publishing the core scientific idea along with a detailed plan on how to put it into practice.

## Catalysts for Change

Once a good number of proposals were open, lots of other changes towards openness would follow across the entire research system.

A natural next step would be to make the peer reviews public [13]—there is little benefit in keeping them secret if the corresponding proposals are public. GMK disagree: publishing “[d]etailed (external) reviews” is the second of the two “radical transparency measures” that they identified in their Box 2 [1]. They state “open access to individual grant review reports may damage reviewers and discourage honest review,” but this seems to assume that reviewer identities were always required to be published along with the reviews, for which I see no necessity. In fact, Copernicus journals have been running public peer review for a decade, and reviewers in their system can still choose to remain anonymous [14]. Why shouldn't this work for grant reviews too? Conversely, mixed reviewing models may speed up the process—why not let proposal authors solicit signed peer reviews from uninvolved authorities in their field and publish them along with the proposal, while inviting the broader community to comment? The small remaining potential for dishonest review can be balanced by classical independent reviews with the option to remain anonymous.

Having proposals and reviews out in the open would allow anyone to consult them when writing or reviewing proposals themselves, and thus help with establishing, maintaining, and teaching quality standards [15],[16]. Other consequences of open proposals could be that rejected proposals could more easily be built upon [17] and that data shown in a proposal might become more likely to actually end up in public databases—and earlier—or that research might be performed more openly, given that the basic ideas are public already. Researchers with a public track record that goes beyond formal publications can eventually be evaluated more on “what they did,” rather than “where they published,” which may mean less proposal writing and more time for research [18].

Data miners could develop tools that highlight assertions in the proposal, link them to the published literature (which would increasingly include proposals) and alert a paper, dataset, proposal, or review when it is cited. Journalists, museums, or other science communicators could begin to interact with research projects before these even start and embed themselves and their audiences into the research process much more than they can now, thereby facilitating new approaches to public engagement with science. Similarly, fellow researchers—or their automated tools—might engage with proposals or the teams behind them in new ways [19]–[21], as exemplified by the Polymath project [22] or the *Escherichia coli* O104: H4 Genome Analysis Crowd-Sourcing Consortium [23], both of which solved complex problems much faster than usual in their domain because of efficient collaboration.

## Conclusions

The article by GMK provides a snapshot of transparency-related practices across major funders of biomedical research and a timely stimulation of debate around this important topic. What I find most illustrative is what none of the surveyed funders publish:

1. assessment summaries,
2. proposals or reviews thereof,
3. information about pending or rejected proposals.

GMK recommend publication in case 1 but not for the other two cases, although they join D&D in inviting experimentation around case 2. I think experimentation has to be encouraged along all three lines and well beyond, since “[i]t would be a fortuitous coincidence if the systems that served us so well in the twentieth century were equally adapted to twenty-first-century needs” [3]. If there are legal barriers to making funding mechanisms more transparent, these have to be addressed in a timely fashion.

According to GMK, funders “must embrace transparency more actively.” To me, this includes research funding mechanisms in general—from individual grants and their peer review up to entire calls and programs—as well as assessing their efficiencies [24]. In particular, “developing countries could leapfrog ahead by adopting from the start science grant systems that encourage innovation” [25], and more systemic transparency may help overcome inequalities in terms of age [26] or other aspects of diversity [27].

It would seem promising to start with publishing successful proposals from the past, along with their reviews, assessment summaries, and final reports. Over time, the embargo period could be reduced, and hopefully, it will eventually vanish. Until then, researchers should be encouraged to share their proposals, reports, and associated reviews early on, and the public to explore these new opportunities for engaging with research.
